# Supplementary material for: The Effectiveness of Learning to Use HMD-Based VR Technologies on Nursing Students: Chemoport Insertion Surgery
Source: Int J Environ Res Public Health. 2022 Apr 15;19(8):4823. doi: 10.3390/ijerph19084823 (PMC9028481; doi:10.3390/ijerph19084823)
Supplement: Supplementary file 1 [file ijerph-19-04823-s001.zip › ijerph-1651269-supplementary.pdf]

Supplementary.

The Effectiveness of Learning to Use HMD-Based VR Technologies on Nursing Students: Chemoport Insertion Surgery

A. General Characteristics

Please check (√) the answer to the question.

1. What is your current age? __________ years

2. What is your gender? ① Male ② Female

3. How satisfied are you with your nursing major?

Very satisfied	Satisfied	Moderate	Not satisfied	Not very satisfied

4. How interested are you in the nursing practice class?

Very satisfied	Satisfied	Moderate	Not satisfied	Not very satisfied

B. Knowledge

The following are questions to determine your knowledge of pre-, during, and post-surgery nursing. Please check(√) where you think is the most correct answer.

1. Where is Chemoport implantation performed?
① neck
② heart
③ abdomen
④ thigh

2. Which blood vessel enters the Chemoport catheter?
① Internal jugular vein
② internal carotid artery
③ Cerebral vein
④ Vertebral vein

3. Where does the Chemoport buried port go?
① neck
② heart

③ chest

④ thigh

4. What is the scope of preparation of the surgical site skin for anticancer drug infusion tube surgery?

① Disinfect from the neck to the abdomen.

② Disinfect the neck and upper chest.

③ Disinfect only the neck.

④ Disinfect only the chest.

5. Where will the surgery be performed?

① Angiography room

② Endoscopy room

③ Outpatient vascular surgery

④ examination room

6. The patient showed anxiety at the entrance to the operating room. Which of the following is the nurse's preoperative response?

① "If you want, we can do a skin transplant."

② "Tell me how you feel before the surgery."

③ "How many patients have these surgeries these days?"

④ "Don't worry, if you get surgery and get chemotherapy, you will get better."

7. The patient returned to the ward after surgery. What should the nurse do first?

① Check the condition by measuring vital signs.

② Reassure the patient that there is nothing wrong.

③ Administer painkillers before complaining of pain.

④ Administer a sedative before waking up from anesthesia and prevent excessive behavior.

8. What device is used to find veins during the Chemoport procedure?

① Doppler

② Ultrasound

③ CT

④ MRI

9. Where is the injection needle placed when administering anticancer drugs?

① wrist

② ankle

③ neck

④ chest

10. How long does the Chemoport procedure take?

① 30 minutes - 60 minutes

② 20 minutes - 40 minutes

③ 15-20 minutes

④ 1 hour or more

C. Learning motivation

The following questions are about feelings or thoughts you felt while learning. Please mark the point that most closely matches your experience or opinion.

	Questionaire	Not at all	←		→	Always
1	When I first started taking classes, I thought it would be easy.	1	2	3	4	5
2	There is something interesting that grabs your attention at the beginning of the lesson.	1	2	3	4	5
3	The material was more difficult to understand than expected.	1	2	3	4	5
4	After listening to or reading the information that guided the class, I felt confident about what I would learn from this class.	1	2	3	4	5
5	I can clearly see to what extent the content of the material is related to what I knew.	1	2	3	4	5
6	It contained so much information that it was difficult to extract or remember important points.	1	2	3	4	5
7	The data caught my eye.	1	2	3	4	5
8	The material showed pictures and examples that people consider important.	1	2	3	4	5
9	Successful completion of class is important to me.	1	2	3	4	5
10	The sentences in the class helped me to concentrate.	1	2	3	4	5
11	The class was so abstract that it was hard to keep my attention.	1	2	3	4	5
12	I was confident that I could know the contents during class.	1	2	3	4	5
13	The class was so much fun that I wanted to know more about the topic.	1	2	3	4	5
14	The class seemed dull and unappealing.	1	2	3	4	5
15	The content of the material was of interest to me.	1	2	3	4	5
16	The way the information was presented helped me stay focused.	1	2	3	4	5
17	There are explanations or examples of how to use knowledge.	1	2	3	4	5
18	There is something in class that stimulates curiosity.	1	2	3	4	5
19	I really enjoyed studying with this class.	1	2	3	4	5
20	A lot of repetition and sometimes boring.	1	2	3	4	5

21	The content and form of the text makes you want to know the content.	1	2	3	4	5
22	I learned something that I was surprised and did not expect from this class.	1	2	3	4	5
23	I am confident that I will be able to pass the exam on this subject after studying in class.	1	2	3	4	5
24	The class is not so relevant to my needs as I already know most of the content.	1	2	3	4	5
25	I felt rewarded for my efforts due to feedback after practice or the language presented in class.	1	2	3	4	5
26	The variety of texts and illustrations helped me to keep my attention in class.	1	2	3	4	5
27	The writing style was boring.	1	2	3	4	5
28	I cannot connect the content of the class with what I have seen or thought in real life.	1	2	3	4	5
29	Too many words on each screen were annoying.	1	2	3	4	5
30	I have a good feeling that I have successfully completed this class.	1	2	3	4	5
31	The content of this class will be useful to me.	1	2	3	4	5
32	I couldn't understand much of the material in this class.	1	2	3	4	5
33	The content was well organized and helped me to feel confident that I would know the material.	1	2	3	4	5
34	It was well designed, so it was fun to study.	1	2	3	4	5

D. Learning attitude

Please indicate the score that corresponds to the question below about your learning attitude when learning.

	**Questionaire**	**Not at all**	**←**		**→**	**Always**
1	I enjoy class time.	1	2	3	4	5
2	I have a lot of different thoughts in class.	1	2	3	4	5
3	I want to learn more about the class.	1	2	3	4	5
4	I make sure to prepare in advance of class.	1	2	3	4	5
5	I study hard only for exams.	1	2	3	4	5
6	I listen intently to lectures in class.	1	2	3	4	5
7	I want to know my score quickly after taking the exam	1	2	3	4	5
8	After class, I organize in my head what I learned in that class.	1	2	3	4	5
9	I think this course is essential to working as a nurse.	1	2	3	4	5
10	I want to do better in this subject than other students.	1	2	3	4	5
11	Even if there is something I don't know in class, I don't ask questions and just move on.	1	2	3	4	5
12	I make plans and work hard to do well in my studies.	1	2	3	4	5

13	I always review what I have learned in class.	1	2	3	4	5
14	I like to give presentations in class.	1	2	3	4	5
15	I am bored with the class time for this subject.	1	2	3	4	5
16	I want to study nursing a lot.	1	2	3	4	5

E. Practice Satisfaction

Please indicate the score that corresponds to the following questions about your satisfaction with practice when you are learning.

	Questionaire	Not at all	←		→	Always
1	I took the practice seriously and participated actively.	1	2	3	4	5
2	Through practice, interest in this field has increased.	1	2	3	4	5
3	The practice atmosphere was good.	1	2	3	4	5
4	The practice contents are logically organized	1	2	3	4	5
5	Practice textbooks and auxiliary materials were appropriate and helpful for learning.	1	2	3	4	5
6	The amount of learning presented in the practice time was appropriate.	1	2	3	4	5
7	The speed of the practice presented in the practice time was appropriate.	1	2	3	4	5
8	The practical contents were interesting.	1	2	3	4	5
9	The practice method was easy to understand.	1	2	3	4	5
10	The materials that lead the practice contents are properly structured.	1	2	3	4	5
11	The current practice method was understood without any help.	1	2	3	4	5
12	The goal to be achieved in the practice has been well accomplished.	1	2	3	4	5
13	Through this practice, I gained the ability to perform actual nursing.	1	2	3	4	5
14	Through this practice, I have developed the ability to communicate with the patient.	1	2	3	4	5
15	Through this practice, the ability to judge a patient's problem has been developed.	1	2	3	4	5
16	I am satisfied with the current practice method	1	2	3	4	5
17	I gained new knowledge through this practice.	1	2	3	4	5
